# Hypoxic Induced Decrease in Oxygen Consumption in Cuttlefish (*Sepia officinalis*) Is Associated with Minor Increases in Mantle Octopine but No Changes in Markers of Protein Turnover

**DOI:** 10.3389/fphys.2017.00344

**Published:** 2017-05-26

**Authors:** Juan C. Capaz, Louise Tunnah, Tyson J. MacCormack, Simon G. Lamarre, Antonio V. Sykes, William R. Driedzic

**Affiliations:** ^1^Centro de Ciências do Mar do Algarve, Universidade do Algarve Faro, Portugal; ^2^Department of Chemistry and Biochemistry, Mount Allison University Sackville, NB, Canada; ^3^Département de Biologie, Université de Moncton Moncton, NB, Canada; ^4^Department of Ocean Sciences, Memorial University of Newfoundland St. John's, NL, Canada

**Keywords:** European cuttlefish, *Sepia officinalis*, HSP70, octopine, polyubiquitinated protein, ventilation frequency

## Abstract

The common cuttlefish (*Sepia officinalis*), a dominant species in the north-east Atlantic ocean and Mediterranean Sea, is potentially subject to hypoxic conditions due to eutrophication of coastal waters and intensive aquaculture. Here we initiate studies on the biochemical response to an anticipated level of hypoxia. Cuttlefish challenged for 1 h at an oxygen level of 50% dissolved oxygen saturation showed a decrease in oxygen consumption of 37% associated with an 85% increase in ventilation rate. Octopine levels were increased to a small but significant level in mantle, whereas there was no change in gill or heart. There were no changes in mantle free glucose or glycogen levels. Similarly, the hypoxic period did not result in changes in HSP70 or polyubiquinated protein levels in mantle, gill, or heart. As such, it appears that although there was a decrease in metabolic rate there was only a minor increase in anaerobic metabolism as evidenced by octopine accumulation and no biochemical changes that are hallmarks of alterations in protein trafficking. Experiments with isolated preparations of mantle, gill, and heart revealed that pharmacological inhibition of protein synthesis could decrease oxygen consumption by 32 to 42% or Na^+^/K^+^ ATPase activity by 24 to 54% dependent upon tissue type. We propose that the decrease in whole animal oxygen consumption was potentially the result of controlled decreases in the energy demanding processes of both protein synthesis and Na+/K+ ATPase activity.

## Introduction

The European cuttlefish (*Sepia officinalis*) is a dominant species in the north-east Atlantic Ocean and Mediterranean Sea. It is a benthic species that occurs to depths of 200 m. Sexually mature and juvenile individuals migrate to coastal grounds in the spring; the latter in pursuit of abundant food sources (Pierce et al., 2008; Bloor et al., 2013). These geographic areas are at risk of becoming hypoxic because of the increase of nutrients and organic matter into the coastal waters (Van Den Thillart et al., 1994; Grantham et al., 2004; Chan et al., 2008). Hypoxic conditions occur naturally in nature (Ekau et al., 2010); however, there are reports of a global oceanic oxygen decline (Schmidtko et al., 2017), which is becoming a problem for marine biodiversity (Vaquer-Sunyer and Duarte, 2008) and an important environment stressor to marine species (Cosme and Hauschild, 2016; Townhill et al., 2017). Hypoxia is also a potential challenge under intensive aquaculture conditions (Brett, 1979; Delaney and Klesius, 2004; Burt et al., 2013) due to the high density of animals and because of the costs associated with keeping oxygen saturation at normal levels. Routine oxygen consumptions of sepioids are within the same range of those of octopus species (Boyle, 1991) and optimal oxygen saturations for *Octopus vulgaris* are reported to be in between 100 and 65% dissolved oxygen saturation (DO_2_) for proper food intake and growth, while suboptimal (modest hypoxia) values range from 65 to 35% DO_2_ (Cerezo Valverde and García García, 2005). These suboptimal values may be reached with a combination of high densities and temperatures in Mediterranean countries, where cuttlefish culture will take place. This is relevant because oxygen availability limits thermal tolerance in *S. officinalis* (Melzner et al., 2006), O_2_ consumption rates rise with increasing temperature up to a metabolic threshold (Melzner et al., 2007) related to the oxygen-binding properties of haemocyanin, which lowers with increasing temperature (Brix et al., 1994). As such, it is important to have an understanding of the response of cuttlefish to challenges of a suboptimal oxygen environment that is considered to be either ecologically and/or practically relevant.

*S. officinalis*, as with other cephalopods, exhibit a decrease in oxygen consumption below a critical environmental oxygen level (Houlihan et al., 1982; Johansen et al., 1982; De Wachter et al., 1988; Cerezo Valverde and García García, 2005; Seibel et al., 2014). Severe hypoxia induces a mobilization of mantle glycogen pools in this species with concomitant accumulation of the anaerobic end product octopine (Storey and Storey, 1979; Storey et al., 1979). The production of octopine is catalyzed by the enzyme octopine dehydrogenase (ODH) that occurs in particularly high levels in mantle, ventricle, and tentacle relative to other tissues (Storey, 1977). Despite this existing information, the biochemical responses of this species, at the tissue level, to environmentally relevant levels of hypoxia have not been fully characterized, so the importance of anaerobic octopine production under these conditions is unclear. In addition to octopine accumulation, more recent studies with the exceptionally hypoxia tolerant Humboldt (jumbo) squid, *Dosidicus gigas*, reveal other aspects of the response to low oxygen levels that include a decrease in mantle contraction frequency (i.e., gill ventilation) and increases in mantle heat shock protein 70 (HSP70) and ubiquinated proteins (Trübenbach et al., 2013a,b, 2014). Here we question if these responses occur in *S. officinalis* as well, as it is considered a hypoxia-sensitive species (Storey et al., 1979).

Decreases in oxygen consumption that are not due to reduced spontaneous swimming activity must be related to decreases in other components of energy demand. Aside from muscle contraction, the two most energy demanding processes in cells are the maintenance of ionic gradients via Na^+^/K^+^ ATPase and protein turnover (synthesis and degradation) (Wieser and Krumschnabel, 2001). The rapid growth rates of cephalopods have been suggested to occur due to high rates of protein synthesis and high efficiency of protein retention, which implies low protein degradation (Houlihan et al., 1990; Carter et al., 2009; Moltschaniwskyj and Carter, 2010). It is possible to measure the relationship between these processes and oxygen consumption by pharmacological inhibition of Na^+^/K^+^ ATPase with ouabain and protein synthesis with cycloheximide (Wieser and Krumschnabel, 2001; Agin et al., 2003). Here we use these pharmacological tools on isolated preparations to assess the potential tissue specific contribution of Na^+^/K^+^ ATPase activity and protein turnover to the whole animal decrease in oxygen consumption.

Mantle is the largest tissue of *S. officinalis*, corresponding to approximately 30–40% of body mass (Castro et al., 1992; Speers-Roesch et al., 2016). We focused our attention on this tissue for assessment of biochemical changes and to determine its potential contribution to alterations in metabolic demand under hypoxia. Although gill and heart represent only about 2 and 0.1% of body mass, respectively (Speers-Roesch et al., 2016), these tissues were also included in our study as they are essential organs to supply oxygen to the rest of the animal, which must maintain function under hypoxia, and have been previously connected to high fractional rates of protein synthesis (Houlihan et al., 1990).

## Materials and methods

### Ethical statement

All the procedures were approved by CCMAR Animal Welfare Committee (ORBEA CCMAR-CBMR) and Direcção-Geral de Alimentação e Veterinária (DGAV) of the Portuguese Government, according to National (Decreto-Lei 113/2013) and EU legislation (Directive 2010/63/EU) on the protection of animals used for scientific purposes. In addition, protocols were approved by institutional Animal Care Committees at each of the Canadian Universities where authors of this paper are based. Procedures were only applied to live animals by authorized users.

### Animals

Cuttlefish (*S. officinalis*) were reared according to the latest culture technology described in Sykes et al. (2014). All experiments used juveniles produced from eggs laid by a F6 captive stock. The mass of group 1 was 59.3 ± 5.4 g (*N* = 18) and of group 2, used only in mantle oxygen consumption experiments to avoid limiting oxygen diffusion across thick mantle tissue, was 1.90 ± 0.14 g (*N* = 7). Experiments were done during May 2016, at CCMAR's Ramalhete Aquaculture Station (Ria Formosa, Portugal—37°00′22.39″N; 7°58′02.69″W). Temperature, salinity, and DO_2_ were measured daily, at 9 h 30, in the stock tank. Both temperature and DO_2_ were measured with a VWR DO220 probe, while salinity was measured with a VWR EC300 salinity meter. Water temperature was 20.5 ± 1.27 (S.D.)°C, salinity was 35.9 ± 0.9 (S.D.) g L^−1^ and DO_2_ level was 101.1 ± 1.2 (S.D.)%. Cuttlefish were fed frozen grass shrimp (*Palaemonetes varians*) *ad libitum* on a daily basis.

### Oxygen uptake and ventilation frequency in whole animals

Two closed respirometry (Lefevre et al., 2016) acrylic chambers (48.5 × 28.5 × 29 cm), each with an external recirculating pump (Maxi-Jet PH MP400) and a PASCO PasPort PPS-2169 multiparameter sensor were used. The recirculating system had a total volume of 41 L. Seawater entered and exited on opposite sides of the chamber at a flow of 200 L h^−1^. Dissolved oxygen saturation data were collected in the upper right section of the chamber at 1 Hz in the PASCO Capstone software and at a temperature of 19.9 ± 1.36 (S.D.) °C. The chamber was completely isolated from any visual stimuli (both researchers and light) by the application of matte black stickers on the outer walls, except from above and one of the 48.5 cm sides. This side had a rectangular LED stripe (50 × 30 × 50 × 30 cm) attached that emitted red light (wavelength peak of 630 nm) until the chamber was completely filled with water and, thereafter, white light (wavelength peaks at 460, 520 and 630 nm) with an intensity of 80 lux. These conditions allowed for the recording of movement (from above) with a Logitech C615 HD camera, and of behavior and ventilation rates (from the side) with a GoPro Hero 3^+^ Black Edition. Video recordings were made for the duration of each experiment at 1,920 × 1,080 pixel resolution and 24 frames per s (fps), from above, and 1,920 × 720 pixel and 50 fps, from the side.

Initially, 6 animals were individually exposed to a DO_2_ of 100% and 6 other animals to a DO_2_ of 50% oxygen saturation for 60 min. Each condition was tested pairwise, 1 cuttlefish at 100% DO_2_ vs. another at 50% DO_2_. At the end of this procedure all the animals were euthanized. Samples of mantle, gill, and heart were rapidly removed and frozen in liquid nitrogen for biochemical analysis. Rates of oxygen consumption ṀO_2_ were determined for animals exposed to 50% DO_2_ during 60 min. In addition, 6 further control animals (DO_2_ of 100%) were used to collect ṀO_2_ during 90 min, to increase the number of available data points for regression fitting. Background ṀO_2_ (resulting from consumption of bacteria and electrode) was also assessed with no animal (*N* = 3) in the chamber for 90 min and was found to be negligible.

Hypoxia at 50% DO_2_ was achieved by gassing seawater with pure N_2_ using a ceramic stone in a closed 90 L reservoir. This water was then injected into the chamber, until the 41 L respirometry recirculating system was filled, and where the animal was already resting. Animals were removed from the stock tank with a black net and transported in a 10 L black bucket filled with seawater to the weighing station. They were individually weighed in a black plastic tray and quickly transported to the chamber area of the experimental room which was illuminated with 1 lux red light conditions (Philips TL-D Colored 18W Red 1SL/25). An outer room containing the data collection stations was isolated from the holding chambers. To avoid inking in the experimental chambers, animals were left resting in the bucket for 5 min before being lightly sedated (mild anesthesia) by adding ethanol to a concentration of 2.5% (Fiorito et al., 2015). After sedation (≈2 min), animals were transferred to the chamber, which had 2 cm in height of seawater and thereafter filled with seawater from the 90 L reservoir (either 100 or 50% DO_2_). As soon as the chamber was filled (≈2 min) and air purged, the system was put in recirculating mode, light switched to white and data acquisition was started on all the probes. This filling time was enough for recovery from sedation at this concentration (Gonçalves et al., 2012).

Ventilation rates were determined from video recordings, according to the following sampling scheme for each animal: (a) first and last 10 min—10 samples of 1 min each; b) from 10 to 50 min in hypoxia and normoxia (control)—1 sample every 10 min. After the procedure, the chamber was emptied, the animal removed, and euthanized with 10% ethanol in seawater (Sykes et al., 2012), the brain was then bisected downwards and forwards, followed by two lateral cuts to sever the brain from the optical lobes (Lewbart and Mosley, 2012). No relevant movements in the chamber nor extreme behavior reactions (Gonçalves et al., 2012) were recorded in any video sampling.

### Biochemical assays

#### Octopine, glucose, and glycogen

Tissues were homogenized and then sonicated (QSonica q55) in 6% HClO_4_ (mantle 1 g tissue: 9 mL HClO_4_; gill 1 g tissue: 4 mL HClO_4_), and subsequently centrifuged at 14,000 × *g* for 10 min at 4°C. The supernatant was neutralized with 2 M KHCO_3_. Octopine was assayed in buffer containing 100 mM TRIS, 8 mM NAD^+^, and excess octopine dehydrogenase (ODH) at pH 9.3. The increase in absorbance at 340 nm, following addition of enzyme, was monitored in a microplate reader. Octopine was calculated based on the NADH extinction coefficient of 6.22 and the pathlength of the plate reader assay was experimentally determined in near IR using the absorbance of water at 975 nm according to the following equation (Lampinen et al., 2012).

$$\begin{array}{l} \text{pathlength}=\frac{OD975_{assay\;buffer}-\;OD900_{assay\;buffer}}{0.173}\times \;10\;mm \end{array}$$

ODH was isolated from frozen scallop, *Placopecten magellanicus*, adductor muscle by a modification of the method of Gäde (1985). Tissue was homogenized (25% w/v) in 100 mM TRIS (pH 7.5, 0.1 mM EDTA, 0.1 mM DTT) and centrifuged at 17,000 × *g* for 10 min at 4°C. Ammonium sulfate was then added to the supernatant up to 65% saturation and stored at 4°C for 7 days to precipitate ODH. The sample was centrifuged, the pellet was re-suspended in 100 mM TRIS (pH 7.5, 0.1 mM EDTA, 0.1 mM DTT), the sample was desalted on a Sephadex G-50 column, and fractions containing ODH activity were retained. Contaminating dehydrogenases were removed by passing the sample through a Cibachrome Blue F3GA agarose column (Affi-Gel Blue, Bio-Rad Laboratories, Hercules, California, USA). Fractions containing ODH activity were combined and subsequently desalted on a Sephadex G-25 column to ensure removal of all residual metabolites. The resulting sample yielded 2 bands on an SDS-PAGE gel, one at ~43 kDa matching the molecular weight of ODH, and a second band of unknown identity at approximately 90 kDa. Lactate dehydrogenase activity was undetectable in the purified sample.

Glucose was analyzed in buffer containing 250 mM imidazole, 5 mM MgSO_4_, 10 mM ATP, 0.8 mM NADP+, excess glucose 6-phosphate dehydrogenase and thereafter treated with excess hexokinase. A standard curve was created for glucose analysis. Assays were conducted in a microplate reader at a wavelength of 340 nm. Glycogen was assessed as glucosyl units following amyloglucosidae treatment of the neutralized supernatant (Keppler and Decker, 1974). Free glucose was subtracted from the total glucosyl equivalents to yield glycogen levels. All organic reagents were purchased from Sigma-Aldrich.

#### Polyubiquitin and HSP70

Polyubiquitinated proteins and HSP70 were determined by western blots. Frozen tissues were sonicated in 9 vol of 50 mM TRIS buffer (pH 7.4) and then centrifuged for 5 min at 14,000 × *g* at 4°C. The protein concentration in the supernatant was determined using the Bradford assay (Bio-Rad) and then adjusted to 3 μg/μL with the homogenization buffer. Tissue extracts were added to Laemmli sample buffer (1:1), boiled for 5 min and 4.5 μg of proteins were resolved by SDS-PAGE (TGX Stain-Free FastCast 10%, Bio-Rad). Resolved proteins were subsequently transferred to PVDF membranes, the membranes were blocked using 5% BSA before being incubated with the primary antibody. To analyze the levels of polyubquitinated proteins, we used an antibody that only recognized proteins containing K48-linked polyubiquitin chains (Abcam ab190061) but had no cross-reactivity to monoubiquitin or polyubiquitin of other linkages. Levels of HSP70 were quantified by loading known quantities of a recombinant HSP70 (Enzo Life Sciences, #ADI-SPP-758, standard levels of 15, 30, and 60 ng per lane) along with the samples and using an HSP70 antibody (#AS05 083A, Agrisera). In all cases, secondary detection was done using an anti-rabbit HRP-linked antibody (for polyubiquitin: #7074, Cell Signaling Technology; for HSP70: SAB-300, Enzo Life Sciences). The chemiluminescence signal was detected using a CCD camera system (ImageQuant LAS 500, GE Life Sciences). Band intensity was quantified using ImageJ (imagej.nih.gov).

### Oxygen uptake by isolated preparations

Tissue ṀO_2_ was measured in 0.20 μm filtered seawater containing an additional 10 mM KCl, 200 mM taurine, and 1 mM glucose. The concentration of taurine used here is representative of measured tissue taurine levels in this species (Maccormack et al., 2016). Cuttlefish were euthanized as described above and 5–10 mg samples of gill, systemic heart, or mantle muscle were collected, weighed, and cut into 2–3 mg pieces using a razor blade. Tissue preparations from the same animal were then transferred to paired respirometry chambers (OX1LP, Qubit Systems Inc., Kingston, ON, Canada) containing 750 μL incubation medium maintained at 20 ± 0.1°C with a recirculating refrigerated water bath. Chambers were calibrated daily and background O_2_ consumption rates were negligible without tissue. Baseline ṀO_2_ was recorded for 300 s before addition of pharmacological agents and recordings were continued for an additional 300 s before experiments were terminated. Treatments consisted of 5 mM ouabain (e.g., Postel et al. (2000)) or 25 μM cycloheximide, a known antibiotic inhibitor of protein synthesis (Giuditta et al., 1968; Prozzo and Giuditta, 1973; Wieser and Krumschnabel, 2001) that has previously been applied in studies associated with learning in the species by blocking protein synthesis (Agin et al., 2003). Stock solutions were prepared in DMSO and delivered to the chambers in 10 μL. A tissue preparation from the same animal was run simultaneously with each drug treatment and exposed to 10 μL DMSO as a vehicle control. Chambers were thoroughly rinsed with 95% ethanol, ddH_2_O, and incubation medium between runs and treatments were alternated between respirometry chambers to control for potential chamber effects. Respirometry chambers were interfaced to a LabQuest data acquisition system and DO_2_ readings were collected over 10 min using LoggerPro V3.8 software (Vernier Software and Technology, Beaverton, OR, USA). Initial tissue ṀO_2_ prior to treatment was determined from the linear decrease in DO_2_ between 120 and 300 s and post-treatment ṀO_2_ was determined between 400 and 600 s. ṀO_2_ was linear with tissue mass (data not shown) and consistent with previous experiments (Maccormack et al., 2016).

### Data analysis and statistics

All values are expressed as mean ± s.e.m with the exception of water temperature, salinity, and DO_2_ that are expressed as mean ± standard deviation. Statistical tests applied are stated in either the Results section or the legends to the figures. All data were tested for normal distribution with the Shapiro-Wilk test as well as for homogeneity of the variances with the Levene's test (Zar, 1999). Statistical difference was considered for *P* < 0.05.

## Results

### Whole animal experiment

**Figure 1:**
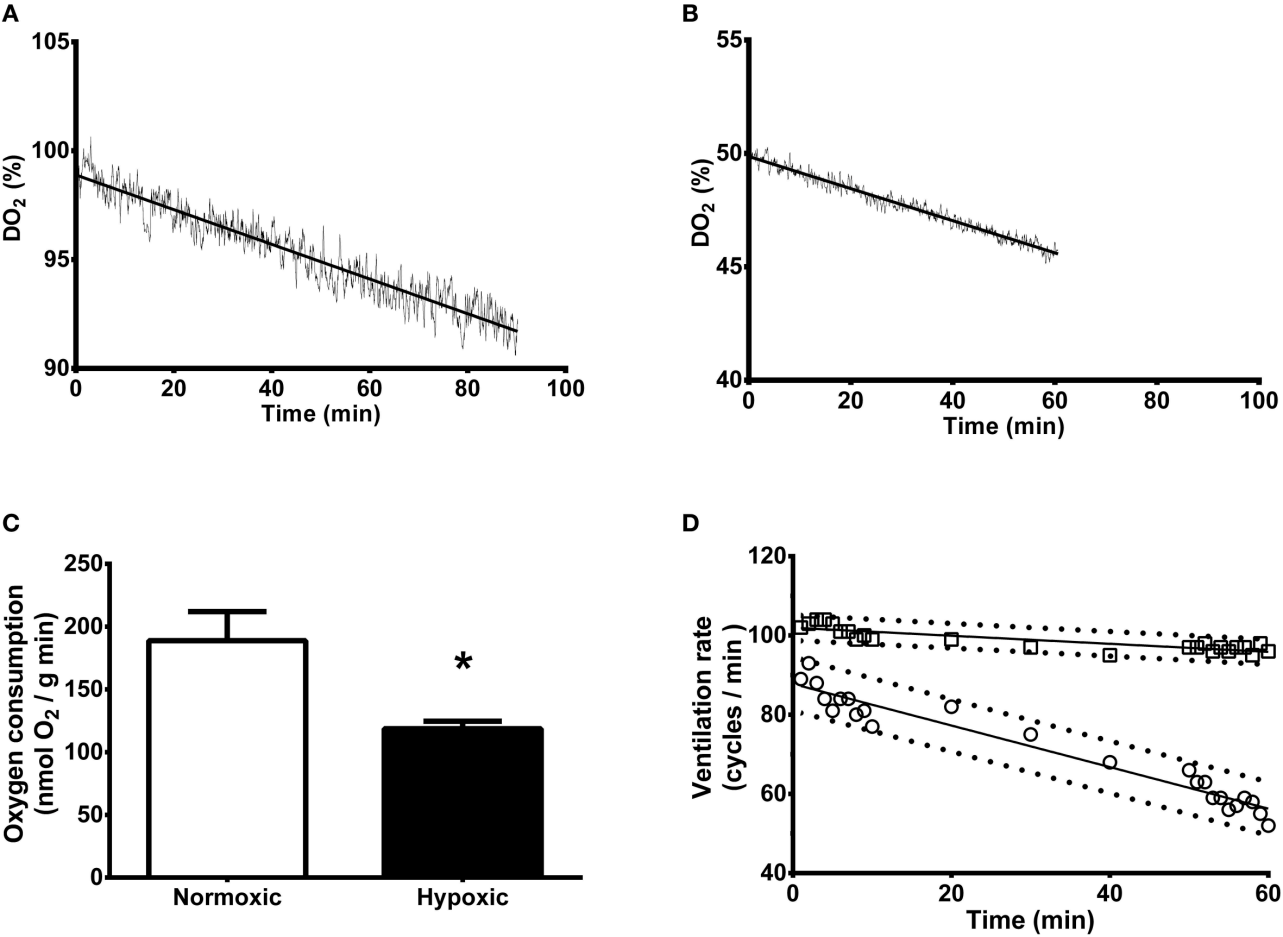
**Whole animal rate of oxygen consumption and ventilation of *Sepia officinalis* under either normoxic or hypoxic (50% dissolved oxygen saturation, 1 h) conditions. (A)** Dissolved oxygen saturation concentration (DO_2_) variation expressed as percentage over a 90 min sampling time in cuttlefish exposed to 100% DO_2_ (control). **(B)** Dissolved oxygen saturation concentration (DO_2_) variation expressed as percentage over a 60 min sampling time in cuttlefish exposed to 50% DO_2_ (mild hypoxia). **(C)** Oxygen consumption expressed as nmol O_2_/(g wet weight animal) min. ^*^Indicates a statistically significant difference between normoxic and hypoxic conditions (2 tailed *t*-test; *P* = 0.025). *N* = 6 for normoxic and *N* = 5 for hypoxic conditions. **(D)** Ventilation rate expressed as cycles/min. Control, circles; Hypoxic, squares. *N* = 4 for all time points. The slopes are significantly different (*P* < 0.001).

Figures 1A,B show examples of dissolved oxygen variation over the sampling period for individual cuttlefish either exposed to 100 or 50% DO_2_ over 90 or 60 min, respectively. Oxygen consumption by hypoxic animals was 37% lower than animals held at 100% air saturation (189 ± 23 vs. 119 ± 6 nmol/g min) (Figure 1C). Cuttlefish maintained under control normoxic conditions exhibited a decrease in ventilation rate over the 60 min holding period (*y* = −0.52x + 88) (Figure 1D). Animals held under hypoxia also displayed a decrease in ventilation rate but to a lesser extent (*y* = −0.1x + 102). After 60 min ventilation rate was 85% higher in hypoxic than normoxic individuals.

**Figure 2:**
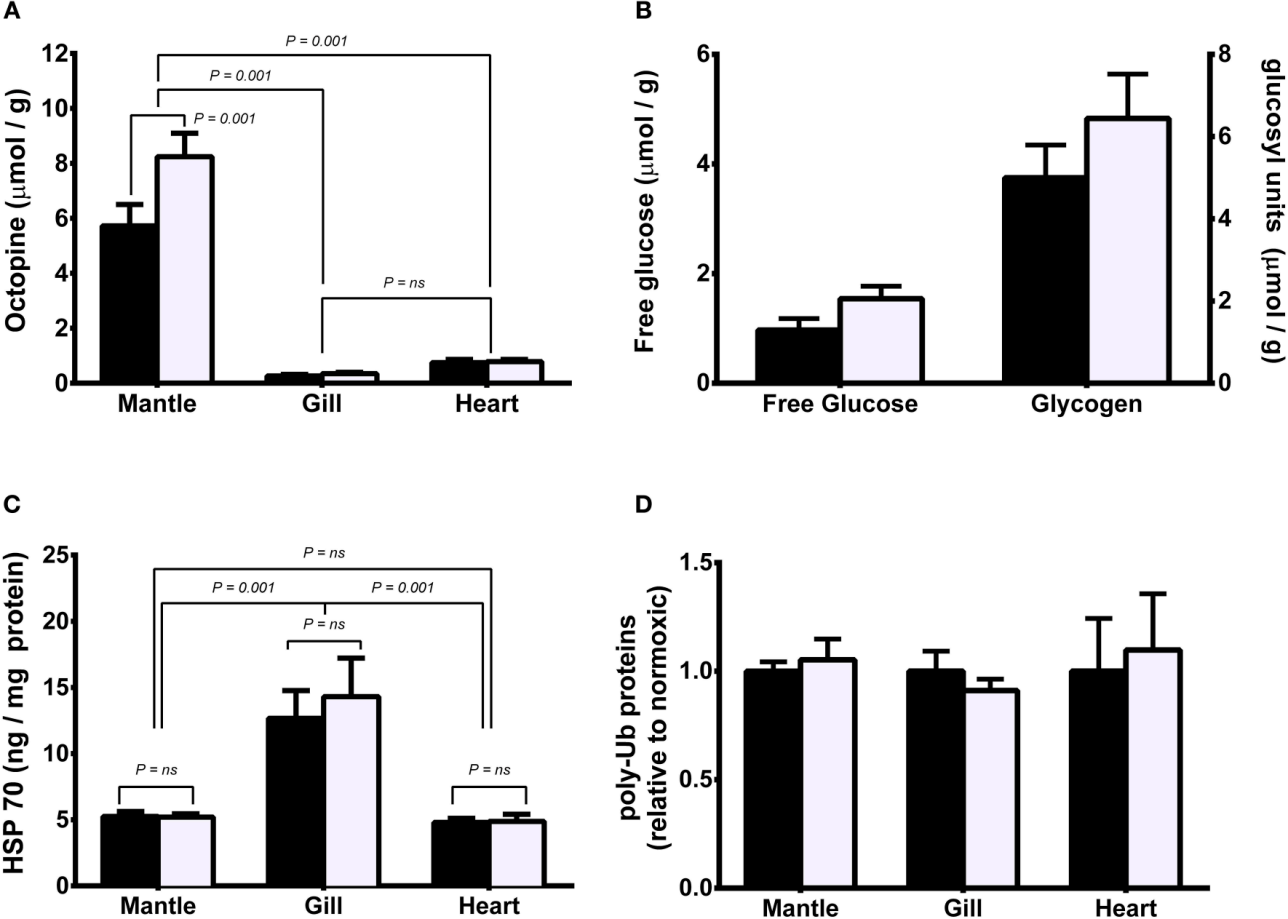
**Metabolite and protein levels in mantle, gill, and heart of *Sepia officinalis* under either normoxic (open bars) or hypoxic (closed bars) (50% dissolved oxygen saturation, 1 h) conditions. (A)** octopine; **(B)** mantle free glucose and glycogen; **(C)** HSP70; **(D)** polyubiquitinated proteins. Statistical significance for octopine, HSP70, and polyubiquitinated proteins, was assessed with a 1-way ANOVA and for differences between glucose or glycogen levels with a *t*-test. *N* = 6 for all conditions except for free glucose in hypoxic mantle where *N* = 4. Differences between means or grouped means represent statistical difference (Tukey's multiple comparison test; *P* < 0.001). No differences were found in mantle free glucose and glycogen nor polyubiquitinated proteins (*P* > 0.05).

Octopine was significantly higher in mantle than in gill or heart regardless of oxygenation condition. Octopine levels were significantly higher in mantle of hypoxic than normoxic animals but there was no significant difference between levels in normoxic or hypoxic animals in either gill or heart. (Figure 2A). There was no significant difference in either free glucose or glycogen levels in mantle between normoxic or hypoxic held animals (Figure 2B). HSP 70 was significantly higher in gill than in mantle or heart regardless of oxygenation condition (Figure 2C). There was no significant difference between levels in normoxic or hypoxic animals in any of the three tissues. Similarly, there was no significant difference in levels of polyubiquinated protein in mantle, gill or heart between treatment groups (Figure 2D).

### Isolated tissue experiment

**Figure 3:**
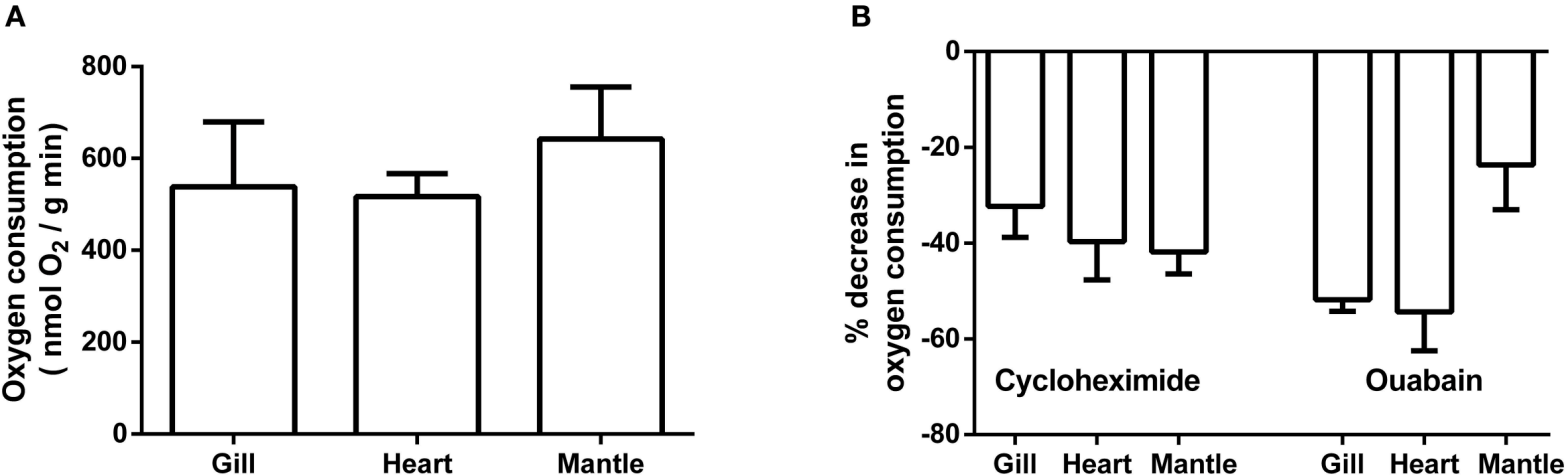
**Basal rate of oxygen consumption and impact of cycloheximide and ouabain on isolated preparations of gill, heart, and mantle from *Sepia officinalis*.** Gill and heart values are from animals of approximately 59 g; while mantle is from animals of ~2 g. **(A)** Rates of oxygen consumption. Statistical significance was assessed with a 1-way ANOVA and no differences were found. *N* = 7 for gill and *N* = 6 for heart and mantle. **(B)** % decrease in oxygen consumption following treatment with cyclohexamide and ouabain *N* = 6 for all experiments. All values were calculated following correction for any impact of DMSO.

Pre-treatment oxygen consumption rates for gill, heart, and mantle are presented in Figure 3A. There was no significant difference in rates amongst the three tissues with values for all being approximately 500 nmol O_2_/gm wet weight. min. Rates for gill and heart are from animals weighing approximately 59 g; while those for mantle are from smaller/younger animals with a mass of approximately 2 g. Preparations were treated with cycloheximide and ouabain to assess how much of the decrease in oxygen consumption noted in the whole animal study could potentially be attributed to protein synthesis and ${\text{Na}}_{/}^{+}$K^+^ ATPase, respectively. Figure 3B shows the percentage decrease in oxygen consumption for each of the tissues following correction for any DMSO effect. Cycloheximide had a substantial effect upon oxygen consumption with average decreases ranging from 32% for gill to 42% for mantle. Similarly, ouabain treatment resulted in average decreases in oxygen consumption from 24% for mantle to 54% for heart.

## Discussion

### Changes in ṀO_2_ and ventilation frequency under hypoxia

Cuttlefish remained sedentary in the respirometry chambers under both normoxic and hypoxic conditions. As such, any difference in oxygen consumption between groups cannot be attributed to spontaneous swimming activity. When corrected for differences in temperature and body mass, the ṀO_2_ values reported here under control conditions are in the same range as rates previously noted (Johansen et al., 1982; Melzner et al., 2006, 2007; Grigoriou and Richardson, 2009; Lamarre et al., 2016). Here, exposure to 50% DO_2_ for 60 min resulted in a 37% decrease in oxygen consumption. Although limited to 2 individuals of *S. officinalis* with a mean mass of 153 g, a decrease in ṀO_2_ of about 29% at a similar level of hypoxia was previously observed by De Wachter et al. (1988). Under comparable conditions, *S. officinalis* of body mass between 100 and 1,500 g showed a decrease in oxygen consumption of approximately 50% (Johansen et al., 1982). It is clear that a substantial reduction in ṀO_2_ is a common response to modest hypoxia in *S. officinalis*; whether life could be sustained indefinitely at this level of hypoxia and if this will impact on the welfare (pain, suffering, distress, and lasting harm) of the animal remains unknown.

Ventilation rate under normoxic conditions decreased over the 60 min holding period in the same fashion as previously shown (Boal and Ni, 1996) and is presumably related to the stress of transferring the animals to the experimental chamber. After 60 min the ventilation rate recorded here was in the same range as previously noted in other studies (Bone et al., 1994; Boal and Ni, 1996; Boal and Golden, 1999; Melzner et al., 2006). Ventilation rate in hypoxic animals remained relatively stable with time and was 85% higher in hypoxic than normoxic cuttlefish after 60 min. To our knowledge this is the first report of rate of ventilation in hypoxic *S. officinalis* or any other cuttlefish species. The response differs from inactive, juvenile Humbolt squid where deep hypoxia resulted in a decrease in ventilation frequency, as well as contraction strength and ventilation volume per min (Trübenbach et al., 2013a). The mantle muscle of squid and cuttlefish consists of a main mass of circular fibers that are mitochondria-poor and primarily anaerobic, as well as inner and outer layers of mitochondria-rich aerobic fibers (Bone et al., 1981; Mommsen et al., 1981). The central fibers are used in escape jetting contractions while the fibers of the inner and outer layers are responsible for rhythmical respiratory ventilation (Bone et al., 1981). It is probable that the increase in ventilation frequency observed here places additional demand to supply ATP to the contractile mechanisms under hypoxia even in the face of reduced oxygen consumption.

### Anaerobic metabolism is minimal

Octopine dehydrogenase is the dominant opine dehydrogenase in *S. officinalis*. We were unable to detect activity of alanopine, tauropine, or strombine dehydrogenase in this species' tissues (unpublished data). The level of ODH in mantle, heart and gill was 35, 20, and 2 μmol/min g wet weight, respectively (Speers-Roesch et al., 2016). The level of octopine in mantle was substantially higher than in heart or gill, consistent with the high activity of ODH in mantle and lower activity in the other two tissues.

Sixty min of 50% hypoxia was associated with an increase in mantle octopine of 2.5 μmol/g. There was no change in octopine level in either gill or heart following the hypoxic period. The observed increase in octopine in mantle was low relative to the capacity to produce this metabolite. For instance, the increase in mantle octopine in *S. officinalis* forced to exercise and then subjected to hypoxia was 13 μmol/g (Storey and Storey, 1979). Another study found a drastic elevation of octopine in the haemolymph of *S. officinalis* exposed to <30% O_2_ hypoxia for about 60 min (Storey et al., 1979). A similar increase in mantle octopine occurred in hypoxic Humbolt squid (Seibel et al., 2014) and squid (*Lolliguncula brevis*) forced to swim to exhaustion (Finke et al., 1996). The modest increase in octopine observed in this study is in line with the lack of change in glycogen levels during the hypoxic challenge. Based on ATP equivalents that may be generated by aerobic and anaerobic metabolism, the contribution of octopine production to overall energy production is minimal.

The relative quantity of polyubiqutinated protein was similar in mantle, gill, and heart under control conditions. There was no impact of the hypoxic challenge in any of the tissues tested. This finding is in contrast to that for juvenile Humbolt squid where severe hypoxia resulted in a three-fold increase in ubiquitinated proteins with a size of approximately 100 kDa (Trübenbach et al., 2014). These authors proposed that the increase in polyubiquintinated protein in association with a decrease in levels of at least two abundant proteins in the same size range (HSP90 and alpha-actinin) provides protection under hypoxia via a number of mechanisms, including the provision of certain amino acids for anaerobic energy production (Trübenbach et al., 2014). In *S. officinalis* food deprivation results in almost total depletion of digestive gland triglyceride, ṀO_2_ decreases by approximately 30%, and mantle polyubiquitin mRNA and polyubiquitinated proteins increase, suggesting an increase in protein degradation via the ubiquitin-proteosome pathway to provide amino acids for energetic purposes (Lamarre et al., 2012, 2016). Although in this species there appear to be mechanisms to increase protein breakdown in association with decreased ṀO_2_, we found no evidence that this occurs under modest hypoxia, at least based on levels of polyubiquinated protein.

HSP70 is a well-recognized stress protein that plays a role in maintaining protein integrity via chaperoning newly synthesized and unfolding proteins (Balchin et al., 2016). HSP70 levels were significantly higher in gill than in mantle or heart and did not change in any tissue with exposure to hypoxia. Relatively high HSP70 levels in *S. officinalis* gill may be related to the high rate of protein synthesis in gill relative to mantle based on direct measurements (Lamarre et al., 2016), and relative to heart and mantle based on total RNA levels (Lamarre et al., 2012). Furthermore, since gill is at the interface with the aquatic environment it may experience more perturbations than other tissues and so has higher constitutive levels of HSPs as protection. HSP70 levels increased in mantle of hypoxic juvenile Humbolt squid (Trübenbach et al., 2013b) but not in adult animals (Seibel et al., 2014). Thus, the generality of a HSP70 response to hypoxia in cephalopods is unresolved but it is clear that under the conditions of the current study there was no change in HSP70 level.

The minimal increase in octopine content, along with stability in glycogen pools, polyubiquitinated protein level, and HSP70 levels in cuttlefish subject to 50% hypoxia for 1 h suggests that anaerobic metabolism is activated to only a limited extent and that the challenge is not particularly stressful to the animal. The 37% decrease in ṀO_2_ must be associated with an equivalent decrease in energy demand. Given that the animals are quiescent under both control and hypoxic conditions, the question becomes as to what metabolic processes are decreased to maintain energy balance.

### Potential sites of energy reduction—insights from isolated tissue experiments

There was no difference in the rate of oxygen consumption between tissue slices of gill, heart, and mantle. The *in vitro* activity of citrate synthase, a qualitative indicator of aerobic metabolism, was approximately 20-fold higher in *S. officinalis* heart than in mantle or gill which were similar to one another (Speers-Roesch et al., 2016). The relatively low rate of oxygen consumption in heart noted here is likely due to the non-contractile nature of the preparation and reflects the basal metabolism of quiescent cells. The heart and gill samples were from juvenile animals and the mantle from hatchlings. Smaller animals have higher mass specific rates of oxygen consumption than larger animals (Johansen et al., 1982; Grigoriou and Richardson, 2009). It is likely that if oxygen consumption of mantle had been conducted on tissue removed from animals the same size as that used for gill and heart that the rate for mantle would have been relatively lower.

One of the major energy demanding processes in non-contracting cells is protein synthesis. In food deprived cuttlefish, both ṀO_2_ and protein synthesis are decreased in concert. A 36% decrease in whole animal ṀO_2_ was associated with a 63% decrease in fractional rate of protein synthesis in both mantle and gill (Lamarre et al., 2016). The importance of these findings in the context of the current study is that it demonstrates that protein synthesis is a regulatable element of the energy demand machinery in *S. officinalis*. Here we show that the inclusion of cycloheximide decreases ṀO_2_ in isolated gill, heart, and mantle preparations. Based on studies with goldfish and trout it is likely that the level of cycloheximide utilized here resulted in a total shut down of protein synthesis (Wieser and Krumschnabel, 2001), as previously suggest by the Agin et al. (2003) results on the species and because the amount used here was more than 3 times higher than the dosages used in *Octopus vulgaris* (Prozzo and Giuditta, 1973) and *Loligo pealii* (Giuditta et al., 1968), which resulted in 80% protein synthesis inhibition in the optic globe with 100 μg/mL and 95–96% inhibition in squid axons with 200 μg/mL, respectively. If it is assumed that all tissues in the quiescent animals are consuming oxygen at similar rates, a total inhibition of all protein synthesis would be required to meet the whole animal decrease in ṀO_2_ of 37%. This is highly unlikely given that even in an anoxic resistant fish, oscar (*Astronotus crassipinnis*), protein synthesis is decreased by only 55% and 60–85% in muscle and liver, respectively, at a DO_2_ of 10% (Lewis et al., 2007). Therefore, although a decrease in protein synthesis could potentially contribute to the decrease in whole animal ṀO_2_, there must be additional mechanisms to reduce energy expenditure.

Na^+^/K^+^ ATPase is a major driving force for many energy dependent ion transport processes and is considered one of the major cellular sites for ATP utilization.

Na^+^ /K^+^ ATPase has been identified in numerous tissues of *S. officinalis* (Donaubauer, 1981) and is presumably ubiquitous. A ouabain sensitive Na^+^/K^+^ ATPase has been identified in gill of squid (*Doritheutis plei*) (Proverbio et al., 1988) and more recently, Na^+^/K^+^ ATPase in *Sepia* gill was shown to increase in response to increases in water CO_2_ level (Hu et al., 2011). In the hypoxia tolerant intertidal clam, *Mercenaria mercenaria*, a severe hypoxic exposure resulted in a decrease in the maximal *in vitro* enzyme activity of Na^+^/K^+^ ATPase (Ivanina et al., 2016). As with protein synthesis, these studies illustrate that Na^+^/K^+^ ATPase is a site that can be controlled to alter energy demand. Here it is shown with isolated tissues that inhibition of Na^+^/K^+^ ATPase with ouabain decreased ṀO_2_ by ~20% in mantle and 50–60% in gill and heart. Given that the Na^+^/K^+^ ATPase is one of the largest consumers of ATP in cells, it is likely that a curtailment of this process occurs in *S. officinalis* under hypoxia.

## Conclusions

A hypoxic challenge to *S. officinalis* of 50% DO_2_ results in only a minor activation of anaerobic metabolism as assessed by octopine accumulation. There were no changes in levels of HSP70 and polyubiquitinated proteins suggesting no alteration in rates of protein breakdown. The animal responds with an increase in ventilation and an overall decrease in metabolic rate, potentially due to decreases in both rates of protein synthesis and Na^+^/K^+^ ATPase activity. The relatively high level of HSP70 in gill compared to mantle and heart is a novel finding and may be related to the high rates of protein synthesis in this tissue.

## Author contributions

SL, TM, AS, and WD participated in the conception and design of research; JC and AS performed the animal husbandry; all authors performed the experiments, acquired and analysed data of work; SL, TM, AS, and WD performed interpretation of data; all authors drafted, edited and approved the final version of the manuscript. All authors agree to be accountable for all aspects of the work in ensuring that questions related to the accuracy or integrity of any part of the work are appropriately investigated and resolved.

### Conflict of interest statement

The handling Editor declared a past co-authorship with one of the authors AS and states that the process nevertheless met the standards of a fair and objective review. The other authors declare that the research was conducted in the absence of any commercial or financial relationships that could be construed as a potential conflict of interest.
