# Corrigendum: Hypoxic Induced Decrease in Oxygen Consumption in Cuttlefish (*Sepia officinalis*) Is Associated with Minor Increases in Mantle Octopine but No Changes in Markers of Protein Turnover

**DOI:** 10.3389/fphys.2019.00018

**Published:** 2019-01-30

**Authors:** Juan C. Capaz, Louise Tunnah, Tyson J. MacCormack, Simon G. Lamarre, Antonio V. Sykes, William R. Driedzic

**Affiliations:** ^1^Centro de Ciências do Mar do Algarve, Universidade do Algarve, Faro, Portugal; ^2^Department of Chemistry and Biochemistry, Mount Allison University, Sackville, NB, Canada; ^3^Département de Biologie, Université de Moncton, Moncton, NB, Canada; ^4^Department of Ocean Sciences, Memorial University of Newfoundland, St. John's, NL, Canada

**Keywords:** European cuttlefish, *Sepia officinalis*, HSP70, octopine, polyubiquitinated protein, ventilation frequency

In the original article, there was a mistake in Figure 2B as published. The right Y-axis of Figure 2B, concerning the amount of glycogen, was incorrectly printed resulting in glycogen levels 10-fold too low. The axis should read “0–8” and not “0–1.” The corrected Figure 2 appears below.

**Figure 2:**
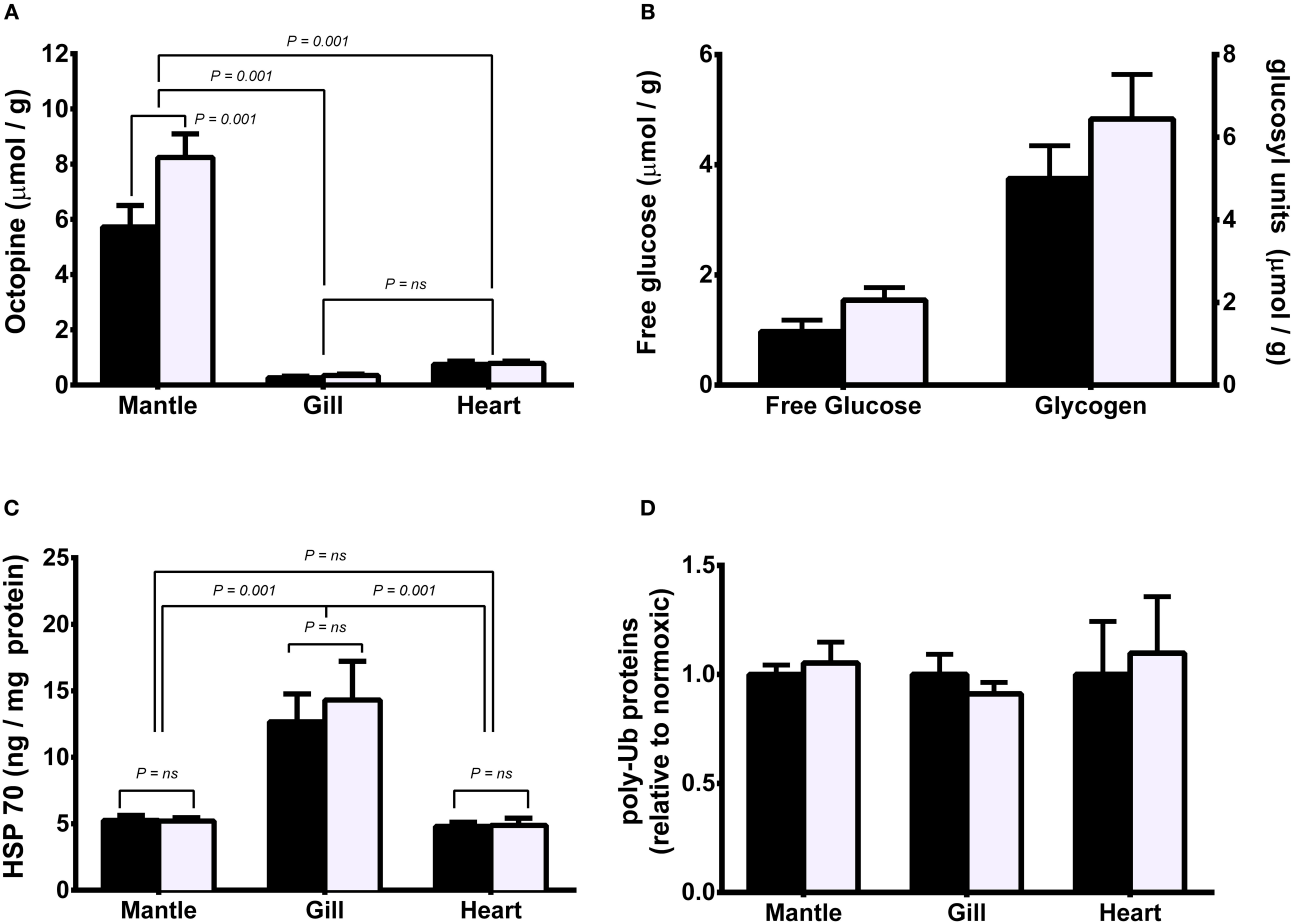
Metabolite and protein levels in mantle, gill, and heart of *Sepia officinalis* under either normoxic (open bars) or hypoxic (closed bars) (50% dissolved oxygen saturation, 1 h) conditions. **(A)** octopine; **(B)** mantle free glucose and glycogen; **(C)** HSP70; **(D)** polyubiquitinated proteins. Statistical significance for octopine, HSP70, and polyubiquitinated proteins, was assessed with a 1-way ANOVA and for differences between glucose or glycogen levels with a *t*-test. *N* = 6 for all conditions except for free glucose in hypoxic mantle where *N* = 4. Differences between means or grouped means represent statistical difference (Tukey's multiple comparison test; *P* < 0.001). No differences were found in mantle free glucose and glycogen nor polyubiquitinated proteins (*P* > 0.05).

The authors apologize for this error and state that this does not change the scientific conclusions of the article in any way. The original article has been updated.

## Conflict of Interest Statement

The authors declare that the research was conducted in the absence of any commercial or financial relationships that could be construed as a potential conflict of interest.

